# The Usefulness of T1-Weighted Magnetic Resonance Images for Diagnosis of Acute Leukemia Manifesting Musculoskeletal Symptoms prior to Appearance of Peripheral Blood Abnormalities

**DOI:** 10.1155/2016/2802596

**Published:** 2016-10-18

**Authors:** Toshihide Yoshikawa, Akihiko Tanizawa, Koji Suzuki, Nanae Tanaka, Taihei Hayashi, Masayo Tsuda, Genrei Ohta, Naoko Kikuchi, Hiroyuki Okamoto, Takehiko Sakai, Yoshihiro Taniguchi, Yusei Ohshima

**Affiliations:** ^1^Department of Pediatrics, Faculty of Medical Sciences, University of Fukui, 23-3 Matsuokashimoaizuki, Eiheiji-cho, Yoshida-gun, Fukui 910-1193, Japan; ^2^Department of Human Resource Development for Cancer, Faculty of Medical Sciences, University of Fukui, 23-3 Matsuokashimoaizuki, Eiheiji-cho, Yoshida-gun, Fukui 910-1193, Japan; ^3^Department of Pediatrics, Japanese Red Cross Fukui Hospital, 2-4-1 Tsukimi, Fukui 918-8011, Japan; ^4^Department of Pediatrics, The Japan Baptist Hospital, 47 Kitashirakawayamanomoto-cho, Sakyo-ku, Kyoto 606-8273, Japan; ^5^Department of Pediatrics, Japan Community Health Care Organization, Kanazawa Hospital, 8-15 Okimachi, Kanazawa 920-0013, Japan; ^6^Department of Pediatrics, Fukui Prefectural Hospital, 2-8-1 Yotsui, Fukui 910-0846, Japan

## Abstract

The patients with acute leukemia occasionally present with musculoskeletal symptoms initially, including bone pain, joint pain, muscular pain, and functional impairment. Without abnormal findings of peripheral blood cell counts or smear, the correct diagnosis tends to be delayed. Magnetic resonance imaging is often performed to examine musculoskeletal abnormalities; it can simultaneously reveal the bone marrow composition with high anatomical resolution and excellent soft tissue contrast. We present 4 pediatric patients who were initially diagnosed with acute pyogenic osteomyelitis or arthritis, based on the elevated white blood cell counts and/or C-reactive protein in addition to the localized high signal intensity on T2-weighted magnetic resonance images. Finally, they were diagnosed with B-cell precursor acute lymphoblastic leukemia by bone marrow examination. The period between the onset of musculoskeletal symptoms and the diagnosis of leukemia ranged from 20 days to 6 months. In all cases, the T1-weighted magnetic resonance images taken prior to detection of peripheral blood abnormality revealed diffuse low signal intensity of the bone marrow in regions adjacent or contralateral to localized musculoskeletal symptoms. These findings should raise the suspicion of leukemia even without abnormalities in peripheral blood.

## 1. Introduction

Abnormal findings of peripheral blood, including the presence of leukemic cells, anemia, and thrombocytopenia, prompt a diagnosis of acute leukemia, although early symptoms of acute leukemia are generally nonspecific, such as fever and fatigue. Musculoskeletal problems, including bone pain, joint pain, muscular pain, functional impairment, and limping, occurred in 20% to 40% of patients with acute leukemia as initial symptoms [1–5]. Patients having musculoskeletal symptoms as the chief complaint sometimes have normal or slightly abnormal peripheral blood findings [1, 6] and these patients are often misdiagnosed with collagen disease or infectious diseases [6]. Although the musculoskeletal pain caused by acute leukemia is described as intermittent, localized, sharp, severe, and of sudden onset and is refractory to treatment with nonsteroidal anti-inflammatory drugs (NSAIDs) [3, 5], the characteristics of the pain are nonspecific. Whenever a second-line treatment with steroids is initiated as an anti-inflammatory or immunosuppressive therapy, it is important to rule out the possibility of acute leukemia, especially acute lymphoblastic leukemia (ALL).

Magnetic resonance imaging (MRI) is relatively noninvasive and is often performed to examine prolonged musculoskeletal problems. It is also the best imaging modality that can directly depict the bone marrow condition and marrow infiltration in malignant diseases, including acute leukemia, with high anatomical resolution and excellent soft tissue contrast [7, 8]. Kato et al. [9] demonstrated that MRI is useful for diagnosing pediatric acute leukemia presenting with prolonged bone pain and without peripheral blood abnormality. In their report, MRI detected leukemic bone marrow involvement by low signal intensity on T1-weighted magnetic resonance images (T1WI) and high signal intensity on T2-weighted magnetic resonance images (T2WI). Here, we present 4 cases with B-cell precursor ALL who had musculoskeletal symptoms and characteristic MRI findings of bone marrow before the appearance of peripheral blood abnormalities. Informed consent was obtained from all patient's parents included in this report.

## 2. Case Presentation

### 2.1. Case  1

**Figure 1:**
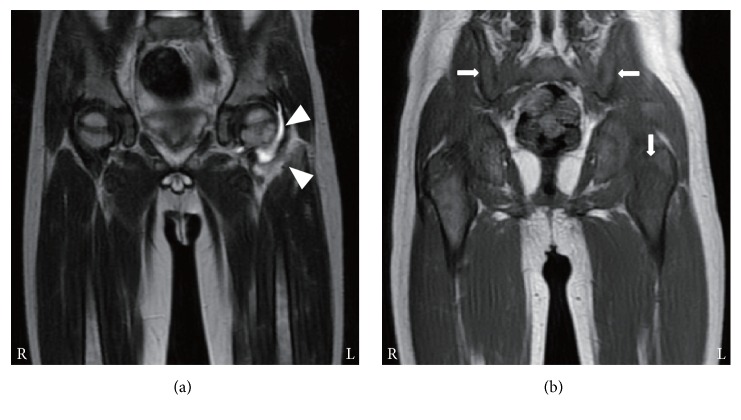
MRI findings in case 1. At 12 days after the disease onset, (a) T2-weighted image shows accumulation of synovial fluid and high signal intensity around the left hip joint (arrowheads) and (b) T1-weighted image depicts low signal intensity areas of the bone marrow in the ilia and the epiphysis of the left femur (arrows).

A 3-year-old boy presented with intermittent high-grade fever, left coxalgia. On the 12th day, he was admitted to a hospital. White blood cell (WBC) counts were slightly elevated and T2WI showed accumulation of synovial fluid and high intensity areas around the left hip joint (Table 1, Figure 1(a)). He was initially diagnosed with acute pyogenic arthritis, but treatment with antibiotics did not produce any clinical response. On the 27th day, the findings of T2WI on second MRI were worsened. Two days later, the histological examination of a bone biopsy specimen obtained from the left femur revealed the presence of bone marrow necrosis and atypical cells. On the 40th day, he was referred to our hospital and bone marrow examination confirmed the diagnosis of ALL. His joint pain immediately disappeared after commencement of chemotherapy.

T1WI obtained on the 12th day had already revealed low signal intensity areas of the bone marrow in the ilia and the epiphysis of the left femur, in contrast to that of the femoral shafts (Figure 1(b)). In this case, an ^18^F-fluorodeoxyglucose (FDG) positron emission tomography/computed tomography on the 42nd day showed the diffuse FDG uptake in the spleen and the bone marrow in the appendicular and axial skeleton including left iliac and femoral bones where T1WI had revealed low signal intensity on the 12th day.

### 2.2. Case  2

**Figure 2 fig2:**
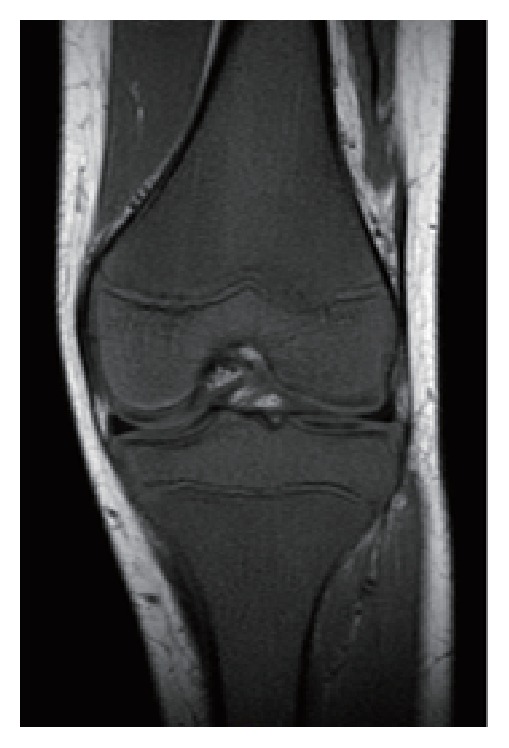
MRI findings in case 2. At 4 months after the disease onset, T1-weighted image depicts low signal intensity in the right femoral and tibial bone marrow.

An 11-year-old boy complained of alternating bilateral knee pain. As the symptom had persisted for 2 months and subsequently shoulder pain occurred, he was examined by an orthopedic surgeon. Physical examination and X-rays demonstrated no remarkable change in these joints. His symptoms spontaneously improved for 2 months. Thereafter right knee pain reappeared. MRI was performed and the images were initially evaluated as normal, although T1WI revealed low signal intensity of the right femoral and tibial bone marrow (Figure 2). Two weeks later, his right knee joint had become swollen. He was admitted to a general hospital for suspicion of juvenile idiopathic arthritis. The C-reactive protein (CRP) level and erythrocyte sedimentation rate were elevated (40 mg/L and 130 mm/hour, resp.), while WBC counts were within the normal range (Table 1). Rheumatoid factor, antinuclear antibody, and anticyclic citrullinated peptide antibody were negative. His joint pain was refractory to NSAIDs treatment; however it was temporarily relieved by intraarticular administration of betamethasone. One and a half months later, he was diagnosed with ALL and referred to our hospital because leukemic cells were detected in the peripheral blood. The joint pain immediately disappeared after chemotherapy.

### 2.3. Case  3

**Figure 3 fig3:**
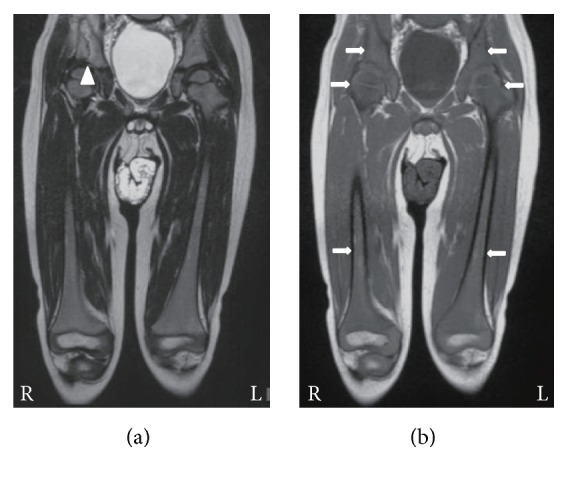
MRI findings in case 3. At 3 days after the onset, (a) T2-weighted image depicts high signal intensity in the right ilium (arrowhead) and (b) T1-weighted images reveal diffuse low signal intensity in the bilateral iliac and femoral bone marrow (arrows).

A 3-year-old boy with high-grade fever and right leg pain was admitted to a hospital on the 3rd day. He was diagnosed as having acute pyogenic osteomyelitis because of elevated WBC counts (10.4 × 10^9^/L), high CRP level (75 mg/L), and high signal intensity of the right iliac bone marrow on T2WI (Table 1, Figure 3(a)). After commencement of antibiotics, his symptoms temporarily improved, but the bilateral leg pain reappeared. On the 20th day, T2WI showed high signal intensity of diaphysis of the right femur and the ilia. The bone biopsy specimens obtained from the right femur and ilium on the 23rd day revealed the presence of bone marrow necrosis and infiltration of inflammatory cells, including neutrophils and lymphocytes. Leukemic cells were detected in peripheral blood the next day and bone marrow examination confirmed a diagnosis of ALL on the 27th day.

T1WI performed on admission had already revealed diffuse low signal intensity of the bilateral iliac and femoral bone marrow (Figure 3(b)).

### 2.4. Case  4

**Figure 4:**
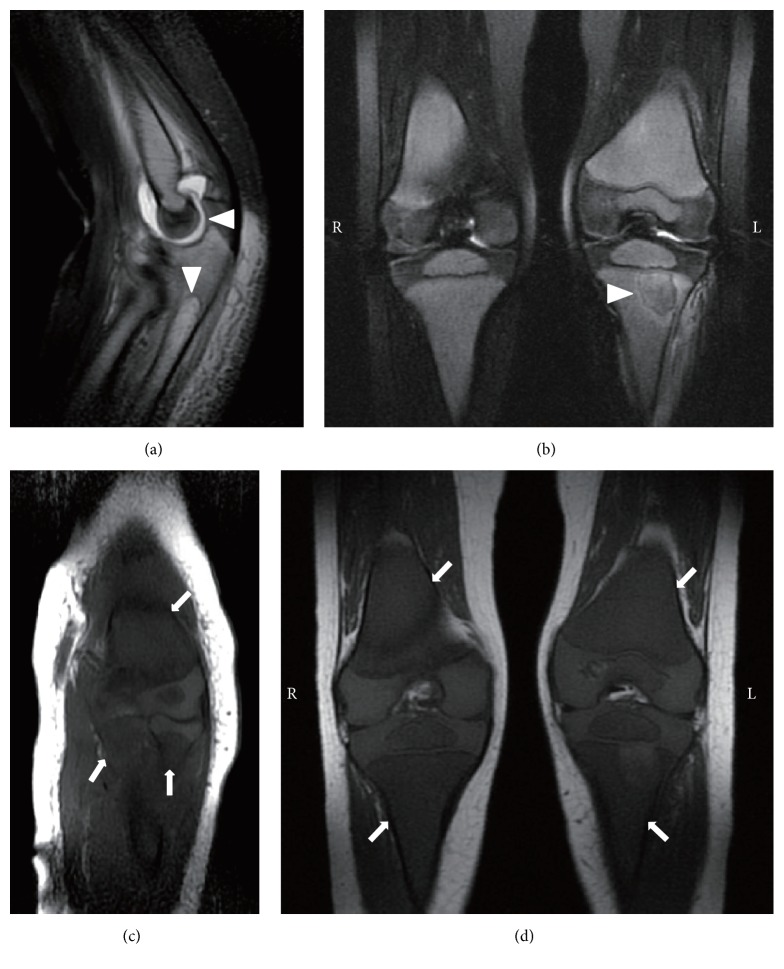
MRI findings in case 4. At 6 days after the disease onset, (a) T2-weighted image shows accumulation of synovial fluid and high signal intensity in the ulnar bone marrow (arrowheads) and (c) T1-weighted images depict diffuse low signal intensity in the left humeral, radial, and ulnar bone marrow (arrows). At 18 days after the onset, (b) T2-weighted image reveals local high signal intensity in the left tibial bone marrow (arrowhead) and (d) T1-weighted image depicts diffuse low signal intensity in the bilateral femoral and tibial bone marrow (arrows).

A 2-year-old boy with fever and left elbow pain and swelling was admitted to a hospital on the 5th day. His blood examination revealed mild neutropenia (1.42 × 10^9^/L) and high CRP level (78 mg/L), and T2WI showed accumulation of synovial fluid and high signal intensity in the ulnar bone marrow (Table 1, Figure 4(a)). After arthrocentesis of the left elbow joint and following administration of antibiotics, the pain diminished and CRP decreased into the normal range. Any bacteria were not isolated by culture of the synovial fluid. On the 18th day, left knee pain occurred and he was diagnosed with osteomyelitis because of high signal intensity on T2WI of the left proximal tibia (Figure 4(b)). On the 20th day, bone marrow examination was performed due to long-lasting neutropenia, and a diagnosis of ALL was confirmed.

T1WI obtained both on admission and on the 18th day had already revealed diffuse low signal intensity of evaluable bone marrow, including the right femoral and tibial regions where the patient had no pain (Figures 4(c) and 4(d)).

## 3. Discussion

Our 4 patients presented with musculoskeletal symptoms and without apparent peripheral blood abnormalities and were initially diagnosed with acute pyogenic osteomyelitis or arthritis. Early diagnosis is difficult when patients manifest musculoskeletal symptoms alone. Indeed, the period between the onset of musculoskeletal symptoms and the diagnosis of ALL ranged from 20 days to 6 months in these patients, as shown in Table 1. High signal intensity on T2WI of the involved bones or joints might lead to misdiagnoses such as acute pyogenic osteomyelitis or arthritis. We demonstrated that T1WI depicted diffuse low signal intensity of bone marrow in regions adjacent to localized musculoskeletal symptoms and on the contralateral side, even early after the onset of ALL (Figures 1 [Fig fig2] [Fig fig3]–4). In case 1, osteolysis was observed on the roentgenogram at the same time when the second MRI was performed, while the other 3 cases had no abnormal X-ray findings.

MRI is a relatively noninvasive and useful modality for visualizing the bone marrow composition and condition, as well as the joints, with high anatomical resolution and excellent soft tissue contrast [7, 8]. In this context, T1WI of bone marrow gives us valuable information to suspect the presence of insidious leukemia. Guillerman [7] describes normal age-related changes in marrow signal intensity on T1WI. In less than 1-year-old infants, the signal intensity of bone marrow on T1WI is low, at the same degree of muscle intensity, in many bones such as femur, sternum, clavicle, humerus, and pelvis. In contrast, in children older than 1 year, when comparing with surrounding muscular intensity, the marrow signal intensity on T1WI has become high in the femur (except for its proximal metaphysis), humerus, and pelvis but still is similar in the sternum and clavicle. Therefore, in children aged above 1 year, diffuse low signal intensity on T1WI of bone marrow in femur, humerus, and pelvic bones suggests a nonphysiological condition.

**Figure 5:**
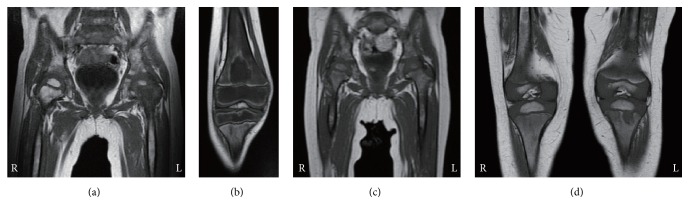
MRI findings after the remission induction chemotherapy. Bone marrow in all cases contains increased signal intensity on T1-weighted images. (a) Case  1, (b) case 2, (c) case 3, and (d) case 4.

Diffuse low signal intensity of bone marrow on T1WI is not specific for acute leukemia and can be seen in other conditions, including myelodysplastic and myeloproliferative syndromes, metastasis of lymphoma, and certain solid tumors [7, 8]. Daldrup-Link et al. [10] reported that bone marrow with decreased cellularity and subsequent fatty conversion after chemotherapy exhibited an increase in signal intensity on T1WI. As shown in Figure 5, consistent with this finding, the signal intensity of involved bone marrow on T1WI partially increased after successful induction chemotherapy in all our cases, suggesting that the initial decreased signal intensity of bone marrow reflected infiltration of leukemic cells in the bone marrow.

It was reported that comorbidity of musculoskeletal problems does not directly affect the prognosis of acute leukemia [2, 4]. However, because appropriate chemotherapy for ALL immediately relieves severe musculoskeletal pain caused by ALL, an accurate early diagnosis is beneficial for these patients. As reported by Kato et al. [9], MRI can detect changes in the bone marrow infiltrated with leukemic cells at an early phase, but local high signal intensity on T2WI might occasionally lead to misdiagnosis with acute osteomyelitis or arthritis. Bone marrow examination is strongly recommended in addition to invasive procedures such as arthrocentesis to the local lesion with high signal intensity on T2WI, whenever T1WI depicts diffuse low signal intensity of bone marrow.

## 4. Conclusion

We should pay attention to the T1WI results of bone marrow in the regions adjacent and/or contralateral to the localized site of musculoskeletal symptoms such as pain. If T1WI depicts diffuse low signal intensity of the bone marrow, even without abnormalities in peripheral blood, this is an important clue to the possibility of acute leukemia in children more than 1 year of age.

## Figures and Tables

**Table 1:** Clinical characteristics of patients.

	Case 1	Case 2	Case 3	Case 4
WBC (×10^9^/L)	11.4	5.1	10.4	5.2
Neutrophil (%)	57.5	60.2	30.2	27.3
Eosinophil (%)	3.0	0.4	0.4	0.6
Basophil (%)	0.5	0.0	0.4	0.2
Lymphocyte (%)	31.0	37.4	61.3	69.6
Monocyte (%)	7.0	2.0	7.7	2.3
Atypical lymphocyte (%)	1.0	0.0	0.0	0.0
RBC (×10^12^/L)	4.55	4.44	4.78	4.28
Hemoglobin (g/L)	127	118	133	118
Hematocrit (%)	35.6	34.6	38.6	34.6
Platelet (×10^9^/L)	366	423	248	364
CRP (mg/L)	10	40	75	78
LDH (IU/L)	336	322	613	261
X-ray findings	Osteolysis	Normal	Normal	Normal
MRI findings				
T1-weighted images	Low	Low	Low	Low
T2-weighted images	High	High	High	High
Interval between the disease onset and MRI analysis	12 days	4 months	3 days	6 days
Initial diagnosis	Pyogenic arthritis	Arthritis	Pyogenic osteomyelitis	Pyogenic arthritis
Duration from the onset to the final diagnosis	40 days	6 months	27 days	20 days
Outcome	1st CR	1st CR	1st CR	1st CR
Follow-up (months)	44	23	104	56

The laboratory data in peripheral blood at an initial medical examination are shown.

WBC: white blood cell; RBC: red blood cell; CRP: C-reactive protein; LDH: lactate dehydrogenase; MRI: magnetic resonance imaging; and CR: complete remission.
